# Identification of *MAVS* as a Novel Risk Factor for the Development of Osteoarthritis

**DOI:** 10.14336/AD.2017.0308

**Published:** 2018-02-01

**Authors:** Jie Liu, Ling-yun Tang, Yan-gui Wang, Shun-yuan Lu, En-ning Zhang, Zhu-gang Wang, Hong-xin Zhang

**Affiliations:** ^1^Shanghai Institute of Orthopaedics and Traumatology, Shanghai Ruijin Hospital, Shanghai Jiao Tong University School of Medicine, Shanghai, 200025, China; ^2^State Key Laboratory of Medical Genomics, Research center for experimental medicine, Shanghai Ruijin Hospital, Shanghai Jiao Tong University School of Medicine, Shanghai, 200025, China; ^3^Department of Clinical Laboratory, Yantaishan Hospital, Yantai, Shandong, 264008, China; ^4^Department of Medical Oncology, Yantaishan Hospital, Yantai, 264000, China

**Keywords:** Osteoarthritis, RIG-I, MAVS, SNP, apoptosis

## Abstract

Evidence indicated that inflammatory response and some pattern-recognition receptors play important roles in the occurrence and progression of osteoarthritis. This study is conducted to evaluate the role of RIG-I and its adaptor protein MAVS in the pathogenesis of osteoarthritis. Four SNPs in *RIG-I* gene and four in *MAVS* gene were genotyped in 1056 Chinese Han population. We also overexpressed MAVS in murine chondrogenic ATDC5 cells and analyzed the cell viability and apoptosis. Rs11795343 (P_-allele_: 0.063394) in *RIG-I*, rs17857295 (P_-allele_: 0.073518) and rs7262903 (P_-allele_: 0.054052, P_-genotype_: 0.067930) in *MAVS* were marginally associated with OA. Rs7269320 (P_-allele_: 0.014783, P_-genotype_: 0.03272) in *MAVS* was significant associated with OA. Further analyses in different genders indicated that rs7262903 (P_-allele_: 0.017256, P_-genotype_: 0.045683) and rs7269320 (P_-allele_: 0.013073, P_-genotype_: 0.038881) are significantly associated with OA in female group. Haplotype analyses indicated G-C-G (χ^2^: 4.328, P_-value_: 0.037503) in rs10813821-rs11795343-rs659527 block of *RIG-I*, G-C-A-T (χ^2^: 4.056, P_-value_: 0.044028) and G-C-C-C (χ^2^: 14.295, P_-value_: 0.000158) in rs17857295-rs2326369-rs7262903-rs7269320 block of *MAVS* were significantly associated with OA. Furthermore, forced expression of MAVS could suppress the viability and promote the apoptosis of ATDC5 chondrogenic cells. In conclusion, this study indicated that RIG-I and MAVS are probably associated with OA in the females of Chinese Han population. And MAVS might be a novel risk factor for OA which may involve in growth of chondrocytes and cartilage homeostasis.

Osteoarthritis (OA) is the most common degenerative disease that affects all the joint components, characterized by progressive loss of articular cartilage, sclerosis of subchondral bone and synovial inflammation [1,2]. It is a major cause of pain, chronic disability in the elderly, and socioeconomic cost across the globe. To date, there is no effective treatment to this disease. Current treatments mainly focus on relieving symptoms and improving function, and joint replacement surgery for end-stage disease [3,4]. Therefore, it is necessary to clarify the pathogenic mechanism underlying this disease, which is the only way to find new effective methods for the prevention and treatment of early osteoarthritis. Previously, osteoarthritis was considered to be a result of “wear and tear” process. In recent years, thanks to numerus clinical researches, it has been regarded as a very complex multifactorial disease resulting from a variety of risk factors including age, gender, ethnicity, daily life and habits, obesity, physical activity, as well as hormonal, mechanical, and genetic factors [5-7]. However, the detailed aetiology of osteoarthritis is still not fully understood. It is well known that articular cartilage plays critical roles in maintaining the structure and function of the joint. The cartilage degradation is considered to be one of the most prominent hallmarks of OA. As the only cell-type in the cartilage, chondrocytes regulate the homeostasis of cartilage. Disturbed chondrocyte behaviors such as apoptosis and/or cellular senescence have been demonstrated to be involved in cartilage degradation. Thus, the risk factors contributing to these processes may play important roles in the pathogenesis of OA. Although the mechanism remains elusive, the relative contribution of chondrocyte apoptosis in the pathogenesis of OA has been well established in numerous studies. Several molecules contributing to chondrocyte apoptosis have been suggested to play important roles in the development of OA. Moreover, because OA is an age-related cartilage degenerative disease, accelerating data has revealed that chondrocyte senescence might ultimately be responsible for the onset of OA. The importance of SA-βgal, caveolin 1 and some other senescence markers in the maintenance and destruction of chondrocyte behavior have been reported by different research groups [8-12].

In the last several years, accelerating evidence indicates that the innate immunity system and inflammatory response play essential roles in the occurrence and progression of osteoarthritis [13-15]. The innate immune system plays a pivotal role in host resistance and tissue homeostasis, which is mediated by pattern-recognition receptors (PRRs). PRRs could sense pathogen-associated molecular patterns (PAMPs) or danger-associated molecular patterns (DAMPs), and then trigger the immune response. Five different classes of PRRs have been identified, including Toll-like receptors (TLR), C-type lectin receptors (CLRs), Nod-like receptors (NLRs), RIG-I (retinoic acid-inducible gene-I)-like receptors (RLRs) and cytoplasmic DNA receptors [16-18]. Recent evidence suggests that some classes of activated PRRs, including TLRs and RLRs, may contribute to the onset and progression of osteoarthritis [19-22]. For example, RAGE (the receptor for the advanced glycation end-products) signaling participates in the development of OA via regulating MMPs and ROS levels [23]. TLR4 signaling also plays pivotal roles in the pathogenesis of OA, and modulating TLR4 signaling in joint tissues is suggested to be a promising target for the therapy of OA [24]. However, some other reports represented controversial results in their studies [25]. Thus, further works are needed to obtain more direct evidence for the participation of PRRs in the pathogenesis of osteoarthritis.

RIG-I (also known as DDX58) is the prototype of the RLR family. Its critical roles in the initiation of anti-viral innate immune responses have been well established. In resting cells, RIG-I is maintained as a monomer in an auto-inhibited state by the repressor domain that functions as an inhibitor. Upon binding to the nonself-ligand RNA in the presence of ATP, RIG-I changes its conformation to expose the CARD (caspase activation and-recruitment domain) domains for signaling [26]. Then the activated Rig-I recruits and activates the downstream mitochondrial antiviral signaling protein MAVS (mitochondrial antiviral-signaling protein; IPS-1/VISA/Cardiff) via CARD-CARD manner, and MAVS subsequently serves as a scaffold that mediates the assembly and activation of the signaling cascades [27]. Radwan and colleagues reported that RIG-I was upregulated in hip cartilage of osteoarthritis patients. They also found that dsRNA-mediated induction of MMP13 required RIG-I, but not MAVS, in chondrocytes. However, the underlying mechanism remains unclear [22].

To further investigate the role of RIG-I and MAVS in the pathogenesis of osteoarthritis, we conducted this study. We totally genotyped and analyzed 4 SNPs (rs10813831, rs10813821, rs11795343, rs659527) of *RIG-I* gene and 4 SNPs (rs17857295, rs2326369, rs7262903, rs7269320) of *MAVS* gene in 602 OA patients and 454 normal controls of Chinese Han origin, and analyzed their association. To further evaluate the potential role of MAVS, we introduced the murine MAVS expression vector into mouse ATDC5 chondrogenic cell line and assessed the cell viability and apoptosis compared with control vectors. To our knowledge, this study is the first to date to evaluate the relationship between the single nucleotide polymorphisms in these two genes and the susceptibility of OA, and the first time to access the role of MAVS in chondrocytes apoptosis and cartilage homeostasis. Our work may provide further insights into the contribution of RIG-I-MAVS signaling related genes in the pathological process of OA.

## MATERIALS AND METHODS

### Study population

The sample set consisted of 602 unrelated osteoarthritis (159 males and 443 females; 64.5±9.5 years old) and 454 normal controls (251 males and 203 females; 58.5±years old) of Chinese Han population recruited from the Department of Orthopaedics of Yantaishan hospital. All patients were diagnosed by senior physicians based on standard clinical, endoscopic, radiologic, and histological criteria, on the basis of two criteria: (1) radiographic evidence of disease (defined as a Kellgren-Lawrence [KL] grade ≧ 2^2^) and/or (2) clinical evidence of disease requiring joint replacement (TJR). Patients with other types of arthritis, skeletal dysplasia or tumor were excluded from the study. Controls were randomly selected from healthy persons under routine health screening. This study was approved by the Research Ethics Committee of Yantaishan Hospital, Yantai, China. And informed consents were obtained from all subjects before blood sampling.

### SNP selection

We consulted the dbSNP (www.ncbi.nlm.nih.gov/snp; accessed October 12, 2012) and HapMap (release #24, CHB; http://hapmap.ncbi.nlm.nih.gov/; accessedOctober 12, 2012) databases and determined the Linkage disequilibrium (LD) block using the criterion of D’ > 0.80 and Haploview version 4.1. LD was computed between every two SNPs to further analyze the haplotype structure. We selected tag SNPs using the software Haploview 4.1, with minor allele frequency (MAF) ≧0.2 and r^2^≧0.5 in the Han Chinese population in Beijing. Finally, we chose 8 SNPs for genotyping (4 SNPs of *RIG-I* gene: rs10813831, rs10813821, rs11795343, rs659527, and 4 SNPs of *MAVS* gene: rs17857295, rs2326369, rs7262903, rs7269320). We tested our SNP variability by using a web tool provided by the Broad Institute (www.broadinstitute.org/mpg/tagger/server.html).

### Genotyping

Genomic DNA was isolated from EDTA peripheral blood using QIAamp blood extraction kit (Qiagen, Hilden, Germany) following the manufacturer’s instructions. All DNA samples were genotyped for *RIG-I* and *MAVS* single nucleotide polymorphisms using allelic specific multiple ligase detection reactions (LDR) according to the standard protocol which were carried out by the Shanghai Generay Biotech Co., Ltd. (www.generay.com.cn/). 10% samples were then confirmed by DNA sequencing to test the validity.

### Plasmid construction

Full-length cDNA of Mouse *MAVS* (NM_144888.2) was amplified by RT-PCR using the forward primer 5′- GAAGGATCCGGTCCGAGTCACTCCAGAAGC-3′ and the reverse primer 5′- AATAAGCTTCCCTGGGCC AGGCGCCTACTACG-3′. The PCR product was sequenced and cloned into pcDNA™3.1/myc-His(-)B vector between the BamHI and HindIII sites. N-terminal Flag-tagged Mouse *MAVS* was cloned into pFLAG-CMV-4 vector between HindIII and BamHI sites, PCR primers used as follow, forward primer 5′-GGGAAGCTTACATTTGCTGAGGACAAGACCTATAAG-3′, reverse primer 5′-AATGGATCCTCACTGGG CCAGGCGCCT ACTAC-3′.

### Cell culture and transfection

The murine ATDC5 chondrogenic cell line were cultured in DMEM/F12 medium containing 10% FBS, 100 U/ml penicillin and 100 µg/ml streptomycin in a humidified incubator at 37ºC containing 5% carbon dioxide. The cells were transfected with pcDNA™3.1/myc-His(-)B empty vector and *MAVS* expressing vector using Lipofectamine 2000 (Invitrogen) according to the manufacturerˊs instructions. 48 hours after transfection, cells were collected for apoptosis assay and western blot analysis.

### Western blot

The cell lysates were prepared by using RIPA buffer (1% Nonidet P-40, 0.5% sodium deoxycholate, 0.1% SDS in PBS) with protease inhibitors (Roche). The protein concentration was assayed by Bradford method, and 20 μg proteins were separated by 10% SDS-PAGE. Standard procedures were used for electrophoresis and immunoblotting analyses. A monoclonal antibodie to Myc-tag (Santa cruz) and a rabbit polyclonal antibody against Gapdh (Sangon) were used as the primary antibodies. The membranes were incubated with IRDyeCW800-conjugated anti-mouse or anti-rabbit immunoglobulin (LI-COR), and scanned with the LI-COR Odyssey imaging system.

### Cell viability assay

The murine ATDC5 chondrogenic cell line were seed in a 96-well plate at a density of 1500 cells/well, and transfected with pcDNA™3.1/myc-His(-)B empty vector and *MAVS* expressing vector, respectively. Cell proliferate ability was evaluated at 0, 1, 2, 3 and 4 days using Cell Counting Kit-8 (Dojindo) according to the protocol of the manufacture. After incubated with CCK8 at 37 ºC for 2 h, the absorbance of each well was measured at 450 nm wavelength using a microplate reader (Biotek).

### Apoptosis analysis

48 hours after transfection, apoptosis of the cells were detected using Annexin V Apoptosis Detection Kit (eBiosicence) following the manufacturer’s instructions. Flow cytometric analyses were carried out on FACSVerse (Becton Dickinson). Data analysis was carried out using FACSuite software (Becton Dickinson).

### Statistical analysis

The differences in the genotype and allele distributions between patients and controls were examined using the χ2 test for independence. In detail, we used Haploview 4.1 to estimate the Hardy-Weinberg equilibrium, linkage disequilibrium, and allelic and haplotype distribution. Hardy-Weinberg equilibrium testing, P-value computations (P > 0.05), in both healthy control and patient groups, odds ratios, allele frequency, and genotypic association were calculated by using SHEsis software (http://analysis.bio-x.cn) [28-30].

## RESULTS

### SNPs test in case-control study

**Figure 1.**
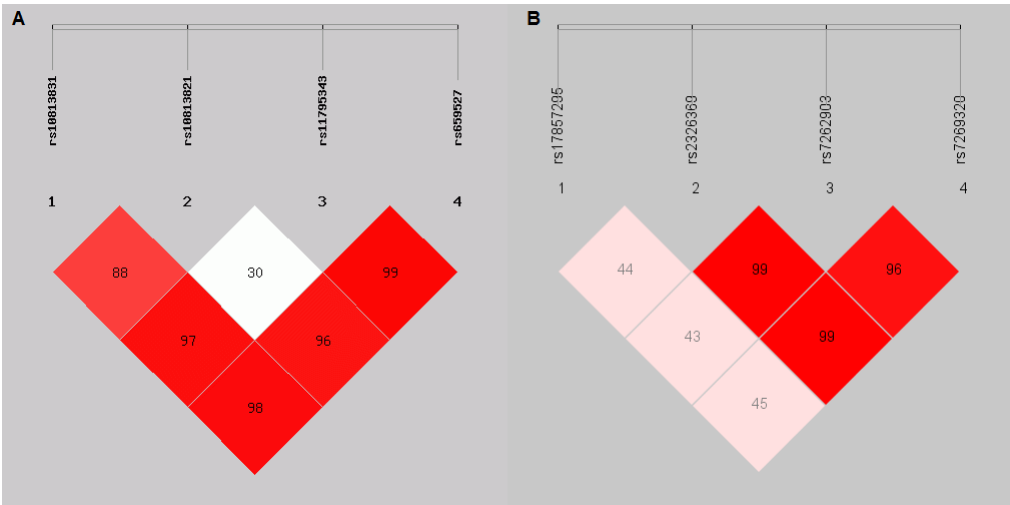
Linkage disequilibrium (LD) block structure consisted of the 8 SNPs located in the two genes, separately (**A**) LD block structure consisted of the 4 SNPs located in RIG-I gene; (**B**) LD block structure consisted of the 4 SNPs located in MAVS gene. The LD block was defined by a D’ value threshold of 0.8. The color scale ranges from red to white (color intensity decreases with decreasing D’ value, and all of D’ values were = 1). This locus was identified as one block, and the plot was generated by Haploview.

The linkage disequilibrium (LD) block structure consisted of the 8 SNPs located in *RIG-I* and *MAVS* genes have been analyzed. (Fig. 1A and 1B) We found that HWE of rs10813831 in *RIG-I* gene is < 0.05, so this SNP was eliminated after the subsequent analyses.

### Allele and genotype analyses in total group

In *RIG-I* gene, rs11795343 showed marginal significant with OA in allele analysis {P_-allele_: 0.063394, OR and [95%CI]: 1.214312[0.989084-1.490828])}. In *MAVS* gene, rs7269320 polymorphism showed significant with OA, in both allele analysis and genotype analysis {P_-allele_: 0.014783, P_-genotype_: 0.03272, OR and [95%CI]: 0.687945[0.508620-0.930495]}, rs17857295 showed marginal significant with OA in allele analysis {P_-allele_: 0.073518, OR and [95%CI]: 1.173689[0.984821-1.398777]}, and rs7262903 showed marginal significant with OA, in both allele analysis and genotype analysis {P_-allele_: 0.054052, P_-genotype_: 0.067930, OR and [95%CI]: 1.337045[0.994199-1.798119]}(Table 1).

### Allele and genotype analyses in female group and male group

In order to find whether gender factor play a role, we subdivided the total subjects to female and male groups. In female group, the results showed that, in *RIG-I* gene, rs11795343 was still marginal significant with OA in allele analysis {P_-allele_: 0.099204, OR and [95%CI]: 1.265292[0.956216-1.674272]}, in *MAVS* gene, rs7262903 and rs7269320 showed significant with OA, both in allele analysis and genotype analysis {rs7262903: P_-allele_: 0.017256, P_-genotype_: 0.045683, OR and [95%CI]: 1.734260[1.097198-2.741217]; rs7269320: P_-allele_: 0.013073, P_-genotype_: 0.038881, OR and [95%CI]: 0.558599[0.350810-0.889462]}. (Table 2) In male group, rs17857295 and rs7269320 polymorphisms in *MAVS* gene showed marginal significant with OA in the allele analyses {rs17857295: P_-allele_: 0.089822, OR and [95%CI]: 1.279793[0.962189~1.702233]; rs7269320: P_-allele_: 0.079560, OR and [95%CI]: 0.675600[0.434962-1.049371]} (Table 3).

**Table 1:** Allele and genotype frequency of the 7 loci in total group.

Genes	SNP ID	Alleles^a^	OR(95%CI)^2^	P-value^1^	Genotypes	HWe P^b^	P-value
*RIG-I*	rs10813821	A(freq) G(freq)			A/A(freq) A/G(freq) G/G(freq)		
	Case	190(0.158) 1014(0.842)	0.958298[0.755065-1.216233]	0.726135	15(0.025) 160(0.266) 427(0.709)		0.480520
	Control	140(0.164) 716(0.836)			16(0.037) 108(0.252) 304(0.710)	0.107796	
	rs11795343	C(freq) T(freq)			C/C(freq) C/T(freq) T/T(freq)		
	Case	316(0.262) 888(0.738)	1.214312[0.989084-1.490828]	0.063394	45(0.075) 226(0.375) 331(0.550)		0.184901
	Control	194(0.227) 662(0.773)			25(0.058) 144(0.336) 259(0.605)	0.405492	
	rs659527	A(freq) G(freq)			A/A(freq) A/G(freq) G/G(freq)		
	Case	723(0.600) 481(0.400)	0.897384[0.749408-1.074579]	0.238849	222(0.369) 279(0.463) 101(0.168)		0.502193
	Control	536(0.626) 320(0.374)			170(0.397) 196(0.458) 62(0.145)	0.651586	
*MAVS*	rs17857295	C(freq) G(freq)			C/C(freq) C/G(freq) G/G(freq)		
	Case	615(0.511) 589(0.489)	1.173689[0.984821-1.398777]	0.073518	167(0.277) 281(0.467) 154(0.256)		0.220791
	Control	403(0.471) 453(0.529)			103(0.241) 197(0.460) 128(0.299)	0.114502	
	rs2326369	C(freq) T(freq)			C/C(freq) C/T(freq) T/T(freq)		
	Case	920(0.764) 284(0.236)	0.930425[0.755102-1.146454]	0.498393	349(0.580) 222(0.369) 31(0.051)		0.697337
	Control	665(0.777) 191(0.223)			259(0.605) 147(0.343) 22(0.051)	0.847191	
	rs7262903	A(freq) C(freq)			A/A(freq) A/C(freq) C/C(freq)		
	Case	137(0.114) 1067(0.886)	1.337045[0.994199-1.798119]	0.054052	12(0.020) 113(0.188) 477(0.792)		0.067930
	Control	75(0.088) 781(0.912)			2(0.005) 71(0.166) 355(0.829)	0.436946	
	rs7269320	C(freq) T(freq)			C/C(freq) C/T(freq) T/T(freq)		
	Case	1066(0.885) 138(0.115)	0.687945[0.508620-0.930495]	0.014783	476(0.791) 114(0.189) 12(0.020)		0.032720
	Control	786(0.918) 70(0.082)			360(0.841) 66(0.154) 2(0.005)	0.578922	

CI: confidence interval; OR: odds ratio p values (p < 0.01) are in italic bold to indicate a trend of significant association. ^1^p-values of the normal chi-square statistics from Monte Carlo stimulation using CLUMP (T2); ^2^OR refers to risk allele odds ratio; Bold numbers represent P-values (P?0.05); ^a^Based on HapMap database release#21; ^b^deviated from Hardy-Weinberg equilibrium.

### Haplotype analyses

Haplotype analyses were also done and the results showed that G-C-G in rs10813821-rs11795343-rs659527 block of *RIG-I* gene was significantly associated with OA {χ^2^: 4.328, P_-value_: 0.037503, OR [95%CI]: 1.288 [1.014~1.635]}. In *MAVS* gene, G-C-A-T and G-C-C-C in rs17857295-rs2326369-rs7262903-rs7269320 block were significantly associated with OA {G-C-A-T: χ^2^: 4.056, P_-value_: 0.044028, OR[95%CI]: 1.440 [1.008~2.057]} {G-C-C-C: χ^2^: 14.295, P_-value_: 0.000158, OR[95%CI]: 0.704 [0.587~0.845], and C-C-C-C was marginal significant with OA {χ^2^: 3.499, P_-value_: 0.061422, OR[95%CI]: 1.202 [0.991~1.459]} (Table 4).

### Reduction of cell growth and induction of cell apoptosis by overexpression of MAVS in murine ATDC5 chondrogenic cell

**Figure 2.**
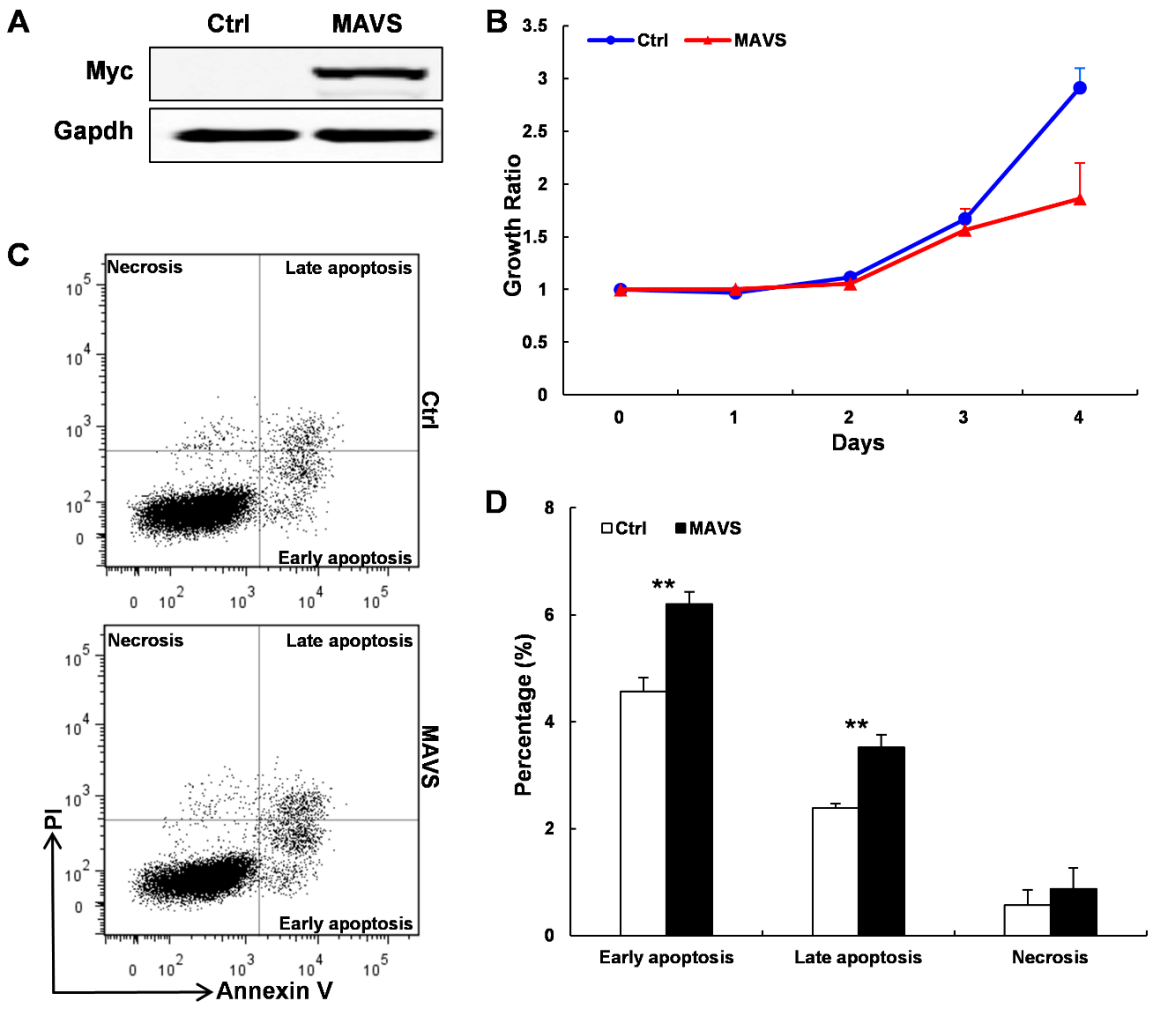
Overexpression of MAVS could suppress viability and promote apoptosis in murine ATDC5 chondrogenic cell (**A**) The proteins expression of MAVS was measured by western blotting. (**B**) CCK-8 cell proliferation assay showed that MAVS overexpression led to decreased cell proliferative capabilities compared with the control cells (P < 0.01). (**C**) Annexin V-FITC/PI staining of ATDC5 cells transfected with MAVS expression vectors as analyzed by flow cytometry. (**D**) The percentages of different cell groups were calculated and expressed as mean ± SD, n = 3 in each group. **P < 0.01 versus control group.

To further evaluate the potential role of MAVS in the pathogenesis of OA, we constructed the expression vectors harboring of murine MAVS. Then the vectors were transfected into murine ATDC5 chondrogenic cell. Successful increases in MAVS expression were measured using western blot (Fig. 2A). The effect of MAVS overexpression in regulating the proliferation of ATDC5 cells was detected by using a CCK-8 cell proliferation assay at baseline and after 1, 2, 3 and 4 days after transfection. As shown in Fig. 2B, MAVS overexpression led to consistently decreased cell proliferative capabilities compared with the control cells (P < 0.01). To analyze the function of MAVS in ATDC5 cell apoptosis, an annexin V/PI staining and FACS assay was used at 48-h post-transfection. The results revealed that over expression of MAVS promotes the spontaneous apoptosis of ATDC5 chondrogenic cells (Fig. 2C, 2D). Considering that MAVS is a C-tail unchored mitochondrial outer membrane protein, we also used the N-terminal flag-tagged MAVS construct to replicate this experiment and got a similar result (Fig. S1). These data indicated that MAVS overexpression result a reduction of cell growth and induction of cell apoptosis in murine ATDC5 chondrogenic cell.

**Table 2:** Allele and genotype frequency of the 7 loci in female group.

Genes	SNP ID	Alleles^a^	OR(95%CI)^2^	P-value^1^	Genotypes	HWe P^b^	P-value
*RIG-I*	rs10813821	A(freq) G(freq)			A/A(freq) A/G(freq) G/G(freq)		
	Case	133(0.150) 753(0.850)	1.062972[0.756771-1.493067]	0.724604	10(0.023) 113(0.255) 320(0.722)		0.348698
	Control	55(0.142) 331(0.858)			7(0.036) 41(0.212) 145(0.751)	0.069465	
	rs11795343	C(freq) T(freq)			C/C(freq) C/T(freq) T/T(freq)		
	Case	241(0.272) 645(0.728)	1.265292[0.956216-1.674272]	0.099204	36(0.081) 169(0.381) 238(0.537)		0.252739
	Control	88(0.228) 298(0.772)			10(0.052) 68(0.352) 115(0.596)	0.989850	
	rs659527	A(freq) G(freq)			A/A(freq) A/G(freq) G/G(freq)		
	Case	534(0.603) 352(0.397)	0.953754[0.746394-1.218722]	0.705020	165(0.372) 204(0.460) 74(0.167)		0.241120
	Control	237(0.614) 149(0.386)			68(0.352) 101(0.523) 24(0.124)	0.148447	
*MAVS*	rs17857295	C(freq) G(freq)			C/C(freq) C/G(freq) G/G(freq)		
	Case	452(0.510) 434(0.490)	1.063286[0.837171-1.350472]	0.614937	124(0.280) 204(0.460) 115(0.260)		0.889261
	Control	191(0.495) 195(0.505)			51(0.264) 89(0.461) 53(0.275)	0.280861	
	rs2326369	C(freq) T(freq)			C/C(freq) C/T(freq) T/T(freq)		
	Case	674(0.761) 212(0.239)	1.009112[0.762890-1.334803]	0.949302	255(0.576) 164(0.370) 24(0.054)		0.989339
	Control	293(0.759) 93(0.241)			111(0.575) 71(0.368) 11(0.057)	0.936182	
	rs7262903	A(freq) C(freq)			A/A(freq) A/C(freq) C/C(freq)		
	Case	95(0.107) 791(0.893)	1.734260[1.097198-2.741217]	0.017256	8(0.018) 79(0.178) 356(0.804)		0.045683
	Control	25(0.065) 361(0.935)			0(0.000) 25(0.130) 168(0.870)	0.335999	
	rs7269320	C(freq) T(freq)			C/C(freq) C/T(freq) T/T(freq)		
	Case	792(0.894) 94(0.106)	0.558599[0.350810-0.889462]	0.013073	356(0.804) 80(0.181) 7(0.016)		0.038881
	Control	362(0.938) 24(0.062)			169(0.876) 24(0.124) 0(0.000)	0.357019	

CI: confidence interval; OR: odds ratio p values (p < 0.01) are in italic bold to indicate a trend of significant association. ^1^p-values of the normal chi-square statistics from Monte Carlo stimulation using CLUMP (T2); ^2^OR refers to risk allele odds ratio; bold numbers represent P-values (P?0.05); ^a^Based on HapMap database release#21; ^b^deviated from Hardy-Weinberg equilibrium

## DISCUSSION

Osteoarthritis is the most common type of joint disease leading to huge socioeconomic problems worldwide. According to the data from WHO, about 9.6% of men and 18.0% of women over 60 years old have symptomatic osteoarthritis, and 25% of those with osteoarthritis cannot perform their major daily activities of life. (www.who.int/chp/topics/rheumatic/en/). Thus, it becomes so important to clarify the pathological mechanism of osteoarthritis. Osteoarthritis is a degenerative disease characterized by progressive loss of articular cartilage, joint space narrowing, sclerosis of subchondral bone and synovial inflammation. Although the detailed etiology of this disease remains poorly elucidated, it is thought to be a multifactorial disease with a complex pathogenesis caused by the interaction of genetic and environmental risk factors [31,32]. During the last decade, studies have indicated that more than 50% of osteoarthritis can be attributed to genetic factors, and a number of OA susceptibility loci have been identified by using genetic studies such as candidate gene studies, linkage studies in multi-case families, twin studies and genome-wide association studies (GWAS) [33-35].

**Table 3:** Allele and genotype frequency of the 7 loci in male group.

Genes	SNP ID	Alleles^a^	OR(95%CI)^2^	P-value^1^	Genotypes^a^	HWe P^b^	P-value
*RIG-I*	rs10813821	A(freq) G(freq)			A/A(freq) A/G(freq) G/G(freq)		
	Case	57(0.179) 261(0.821)	0.989182[0.682903-1.432825]	0.954100	5(0.031) 47(0.296) 107(0.673)		0.921633
	Control	85(0.181) 385(0.819)			9(0.038) 67(0.285) 159(0.677)	0.562933	
	rs11795343	C(freq) T(freq)			C/C(freq) C/T(freq) T/T(freq)		
	Case	75(0.236) 243(0.764)	1.059865[0.756359-1.485159]	0.735528	9(0.057) 57(0.358) 93(0.585)		0.759756
	Control	106(0.226) 364(0.774)			15(0.064) 76(0.323) 144(0.613)	0.255147	
	rs659527	A(freq) G(freq)			A/A(freq) A/G(freq) G/G(freq)		
	Case	189(0.594) 129(0.406)	0.837909[0.625558-1.122345]	0.235425	57(0.358) 75(0.472) 27(0.170)		0.304381
	Control	299(0.636) 171(0.364)			102(0.434) 95(0.404) 38(0.162)	0.052084	
*MAVS*	rs17857295	C(freq) G(freq)			C/C(freq) C/G(freq) G/G(freq)		
	Case	163(0.513) 155(0.487)	1.279793[0.962189~1.702233]	0.089822	43(0.270) 77(0.484) 39(0.245)		0.239040
	Control	212(0.451) 258(0.549)			52(0.221) 108(0.460) 75(0.319)	0.269945	
	rs2326369	C(freq) T(freq)			C/C(freq) C/T(freq) T/T(freq)		
	Case	246(0.774) 72(0.226)	0.900090[0.637949-1.269947]	0.548880	94(0.591) 58(0.365) 7(0.044)		0.696490
	Control	372(0.791) 98(0.209)			148(0.630) 76(0.323) 11(0.047)	0.756956	
	rs7262903	A(freq) C(freq)			A/A(freq) A/C(freq) C/C(freq)		
	Case	42(0.132) 276(0.868)	1.278261[0.825347-1.979714]	0.270496	4(0.025) 34(0.214) 121(0.761)		0.363315
	Control	50(0.106) 420(0.894)			2(0.009) 46(0.196) 187(0.796)	0.650864	
	rs7269320	C(freq) T(freq)			C/C(freq) C/T(freq) T/T(freq)		
	Case	274(0.862) 44(0.138)	0.675600[0.434962-1.049371]	0.079560	120(0.755) 34(0.214) 5(0.031)		0.148153
	Control	424(0.902) 46(0.098)			191(0.813) 42(0.179) 2(0.009)	0.852830	

CI: confidence interval; OR: odds ratio. ^1^p-values of the normal chi-square statistics from Monte Carlo stimulation using CLUMP (T2); ^2^OR refers to risk allele odds ratio; ^a^Based on HapMap database release#21; ^b^deviated from Hardy-Weinberg equilibrium.

In this study, we initial genotyped 4 SNPs in *RIG-I* gene and 4 in *MAVS* gene. For the reason that HWE value of rs10813831 in *RIG-I* was minor than 0.05, we discarded this SNP and finally selected the other 7 polymorphisms during the following tests. The results indicated that both *RIG-I* and *MAVS* genes might play important roles in OA in Chinese Han Population, especially the latter. In the total group, *RIG-I* was marginal significant associated with OA, and *MAVS* was significant associated with this disease. After being subdivided into male group and female group, the trend stayed the same. Furthermore, the haplotype results still supported the trend above. The results showed that G-C-G haplotype in the block of rs10813821-rs11795343-rs659527 in *RIG-I* gene, and C-C-C-C haplotype in the block of rs17857295-rs2326369-rs7262903-rs7269320 in *MAVS* gene were marginal significant. Besides, in rs17857295-rs2326369-rs7262903-rs7269320 block of *MAVS*, G-C-A-T and G-C-C-C were positively associated with OA. These results of our case-control study have thrown a light on the important role of *RIG-I* and *MAVS* genes in the OA. Besides, the results indicated that there is some relationship between *RIG-I* and *MAVS* genes.

MAVS is a mitochondrial transmembrane protein that contains a N-terminal caspase activation and -recruitment domain (CARD) and a C-terminal transmembrane domain (TM). It has been demonstrated that MAVS plays a central role in bridging the interaction between RLRs and downstream effectors during anti-viral innate immune response [36,37]. However, emerging evidence indicates that MAVS is essential in cell death signaling. The ectopic MAVS expression led to significant reduction in the cell viability and induction of cell apoptosis in a dose-dependent manner [38,39]. To further explore the physiological role of MAVS in the development of osteoarthritis, we introduced the murine MAVS expression vector into in the mouse chondrogenic cell line ATDC5. As expected, transient MAVS expression in ATDC5 cells resulted in a significant loss of cell viability and marked induction of apoptosis compared with the control cells. Several studies have shown that changes in chondrocyte behavior, such as enhancement of chondrocyte apoptosis and necrosis were involved in the pathogenesis of OA. Although more precise work is needed to find the functional role of MAVS playing in the homeostatic control of chondrocyte, our data indicate that MAVS disrupts cartilage homeostasis and promotes the progress of OA by enhancing the apoptosis of chondrocytes.

**Table 4:** Haplotype analysis.

Group	Haplotype	Case(freq) (%)	Control (freq)(%)	χ^2^	P	OR[95%CI]
rs10813821-rs11795343-rs659527	A C A	0.00(0.000)	1.37(0.002)	-	-	-
	A C G	95.49(0.079)	66.27(0.077)	0.017	0.89755	1.022 [0.737~1.416]
	A T A	1.34(0.001)	3.37(0.004)	-	-	-
	A T G	93.17(0.077)	68.99(0.081)	0.088	0.767199	0.952 [0.688~1.317]
	G C G	220.51(0.183)	126.36(0.148)	4.328	0.037503	1.288 [1.014~1.635]
	G T A	721.66(0.599)	531.26(0.621)	1.210	0.271267	0.904 [0.755~1.082]
	G T G	71.83(0.060)	58.38(0.068)	0.658	0.417168	0.863 [0.604~1.233]
rs17857295-rs2326369-rs7262903-rs7269320	C C A C	0.41(0.000)	2.98(0.003)	-	-	-
	C C A T	36.50(0.030)	19.31(0.023)	1.118	0.290300	1.350 [0.772~2.361]
	C C C C	380.31(0.316)	236.89(0.277)	3.499	0.061422	1.202 [0.991~1.459]
	C T A C	0.00(0.000)	0.14(0.000)	-	-	-
	C T A T	0.00(0.000)	0.03(0.000)	-	-	-
	C T C C	197.49(0.164)	143.65(0.168)	0.067	0.796321	0.969 [0.766~1.227]
	G C A C	3.60(0.003)	3.90(0.005)	-	-	-
	G C A T	96.37(0.080)	48.63(0.057)	4.056	0.044028	1.440 [1.008~2.057]
	G C C C	397.79(0.330)	351.28(0.410)	14.295	0.000158	0.704 [0.587~0.845]
	G C C T	4.74(0.004)	2.02(0.002)	-	-	-
	G T C C	86.39(0.072)	47.17(0.055)	2.236	0.134787	1.321 [0.916~1.906]
	C C C T	0.27(0.000)	0.00(0.000)	-	-	-
	G T A T	0.11(0.000)	0.00(0.000)	-	-	-
rs2583760-rs2583759 Global result:						
Global chi2 is 156.695374 while df=1 (frequency<0.03 in both control & case has been dropped.)				
Pearson’s p value is 0.00E+000						
Permutation p value(Pearson) is 0.0000						
rs2583764-rs2583760-rs6993386-rs2583759 Global result:					
Global chi2 is 291.831390 while df=5 (frequency<0.03 in both control & case has been dropped.)				
Pearson’s p value is Pearson’s p value is 0.00E+000						
Permutation p value(Pearson) is 0.0000						

Haplotypes observed in <1% of the control subjects are not listed in the table. OR: odds ratio; the OR in one block for each haplotype was calculated by using all the other haplotypes in the same block as the reference haplotype. Significant P values (P < 0.05) are in bold.

On the other hand, there are several potential limitations of our study which should be declared here. Firstly, the patients and controls in our study were all from Yantai area. So, multi-center studies based on samples from different regions would give us more convincing results. Secondly, the relative small sample size in the male group may have reduced the power of the statistical analyses. And absence of assessment on the relationship between gene expression levels and development of OA might be another limitation. In spite of these limitations, our study represents the first attempt to evaluate the potential role of RIG-I and MAVS in the pathogenesis of OA. The data indicated that RIG-I and MAVS are probably associated with OA, and MAVS might be a risk factor for OA in the Chinese Han population, especially in the females. The ectopic MAVS expression caused chondrocytes apoptosis might be responsible for the development of osteoarthritis.

Although replications based on independent samples of further studies are necessary, the current study provides some new insights into the relationship between innate immune related molecules and the pathogenesis of OA. All of these are absolutely useful in better understanding the underlying biology of this complex disease. Our present work and, hopefully, follow-up studies could shed more light on the effects of innate immune signaling cascade in chondrocytes behavior and cartilage homeostasis. And all of these will be much important for the prevention, diagnosis and therapy of osteoarthritis.

## 

Figure S1 N-terminal flag-tagged MAVS construct promote apoptosis in murine ATDC5 chondrogenic cell.
