# Supplementary figures and images for: Identification of MAVS as a Novel Risk Factor for the Development of Osteoarthritis
Source: Aging Dis. 2018 Feb 1;9(1):40–50. doi: 10.14336/AD.2017.0308 (PMC5772857; doi:10.14336/AD.2017.0308)

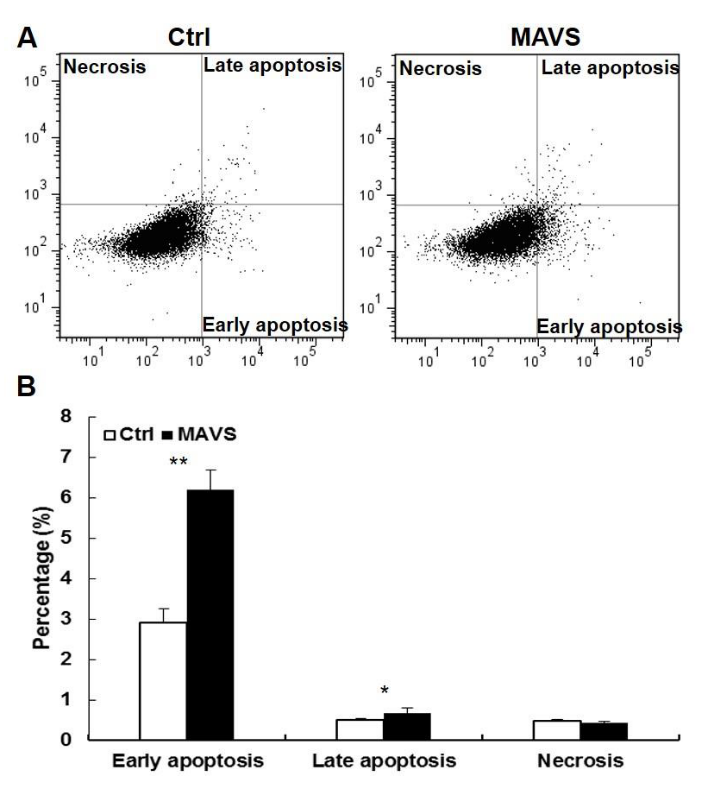

Supplement: Figure S1 — N-terminal flag-tagged MAVS construct promote apoptosis in murine ATDC5 chondrogenic cell. [file ad-9-1-40-g3.tif]
